# Discovery of benzo[e]pyridoindolones as kinase inhibitors that disrupt mitosis exit while erasing AMPK-Thr172 phosphorylation on the spindle

**DOI:** 10.18632/oncotarget.4158

**Published:** 2015-06-11

**Authors:** Ly-Thuy-Tram Le, Morgane Couvet, Bertrand Favier, Jean-Luc Coll, Chi-Hung Nguyen, Annie Molla

**Affiliations:** ^1^ INSERM UJF U823 Institut Albert Bonniot, Team 5, BP 170, Grenoble Cedex 9, France; ^2^ Department of Biotechnology, University of Sciences and Technology, DaNang, Vietnam; ^3^ Université Joseph Fourier - Grenoble, Team GREPI, Etablissement Français du Sang, BP35, La Tronche, France; ^4^ Institut Curie, PSL Research University, UMR 9187 – U 1196 CNRS-Institut Curie, INSERM, Bat 110 Centre Universitaire, Orsay, France

**Keywords:** mitosis, aurora kinase, kinase inhibitor, cancer therapy

## Abstract

Aurora kinases play an essential role in mitotic progression and are attractive targets in cancer therapy. The first generation of benzo[e]pyridoindole exhibited powerful aurora kinase inhibition but their low solubility limited further development. Grafting a pyperidine-ethoxy group gives rise to a hydrosoluble inhibitor: compound C5M.

C5M could efficiently inhibit the proliferation of cells from different origins. C5M prevented cell cycling, induced a strong mitotic arrest then, cells became polyploid and finally died. C5M did not impair the spindle checkpoint, the separation of the sister chromatids and the transfer of aurora B on the mid-zone. C5M prevented histone H3 phosphorylation at mitotic entry and erased AMPK-Thr172 phosphorylation in late mitosis. With this unique profile of inhibition, C5M could be useful for understanding the role of phospho-Thr172-AMPK, in abscission and the relationship between the chromosomal complex and the energy sensing machinery.

C5M is a multikinase inhibitor with interesting preclinical characteristics: high hydro-solubility and a good stability in plasma. A single dose prevents the expansion of multicellular spheroids. C5M can safely be injected to mice and reduces significantly the development of xenograft. The next step will be to define the protocol of treatment and the cancer therapeutic field of this new anti-proliferative drug.

## INTRODUCTION

Aurora kinases are a family of serine/threonine protein kinases that play key roles in mitotic progression [1, 2]. In humans, three aurora kinases have been identified: A, B, and C. Aurora A is initially associated with centrosomes and then with spindle microtubules, whereas aurora B is a chromosomal passenger protein travelling from centromeres to microtubules with its partners: survivin, INCENP and borealin. Aurora A is required for centrosome duplication, entry into mitosis, formation of bipolar spindles, and mitotic checkpoints [3, 4]. Aurora B is essential for chromosome condensation, kinetochore functions, spindle checkpoint activation, and cytokinesis completion [2, 5]. Aurora C is involved in spermatogenesis and was recently also described in mammalian oocytes where it efficiently executed meiosis I and ensured high-quality eggs necessary for sexual reproduction [6].

Aurora kinases A and B are overexpressed in many cancers, including primary colon and breast cancers [7, 8]. In light of these observations, aurora kinases have emerged as potential druggable targets for anticancer therapy, and many small molecule inhibitors of aurora kinases have been developed [9, 10]. Several of these ATP-competitive inhibitors are currently in clinical development [11, 12], [https://clinicaltrials.gov/ct2/results?term=%22aurora+kinase%22]. Preliminary data from clinical trials generally indicate disease stabilisation, with the best responses achieved in patients suffering from refractory chronic myeloid leukemia [13].

According to their respective role in mitosis, specific aurora A inhibitors seemed the most promising since they induced the death of mitotic cells. Aurora B kinase inhibition induced mitotic exit and generated genomic instability [14]. However the best treatment strategy remains an open question. To date, no class has been superior in early clinical trials. In phase I studies of solid tumours, treatment with Aurora A kinase inhibitor alisertib resulted in only one partial response, although stable disease was reported in other patients [15, 16]. Similar results were seen with the selective aurora B kinase inhibitor barasertib, with no responses observed in advanced solid tumours, in a phase I study [17]. Like alisertib, barasertib showed more promising clinical activity in hematologic malignancies, with a response rate of 25% in patients with newly diagnosed or relapsed acute myeloid leukemia [18]. Till now none of these drugs was approved for clinical use. A careful selection of the patient population may be critical for achieving favourable therapeutic outcomes. Furthermore promising results were observed when simultaneously inhibiting aurora and off-targets like mutated bcr-abl or in synergy with cancer drugs or radiotherapy. In conclusion, the aurora kinase inhibitors could not have broad applications in cancer therapy. Their potential could be evaluated through a description of the mode of action and will depend on tumour markers.

In a previous study, we identified benzo[e]pyridoindolones (BePI) as novel potent inhibitors of the aurora kinases. The best hit, compound 1, is an ATP-competitive inhibitor with nanomolar *in vitro* aurora kinase inhibitory activity [19, 20] and sensitizes glioma stem cells to radiation treatment [21]. However, its use was limited by its low solubility in water. A structure/activity study opened the possibility to propose hydrosoluble BePI with high antiproliferative activity [22]. An amino-ethyl chain engrafted on the scaffold allowed us to prepare the water-soluble salt thereof. The aim of the present study was to define the best amino chain preserving aurora B inhibition and to describe its cellular effects. C5M with a pyperidine-ethoxy group exhibited the highest aurora B inhibition, *in vitro*. We showed that C5M could efficiently prevent proliferation of cells at about 100 nM. Actually, C5M prevented cell cycling, induced a strong mitotic arrest then, cells became polyploid and finally died. Conversely to compound 1, C5M did not impair the spindle checkpoint, the segregation of daughter chromatids and the transfer of aurora B on the mid-zone. The mitotic drug C5M was found to be a multikinase inhibitor, with restraint selectivity, that prevented histone H3 phosphorylation at mitotic entry and erased AMPK-Thr172 phosphorylation in late anaphase. These are unique features for aurora B kinase inhibitors. Moreover C5M has interesting preclinical characteristics and prevents tumour growth both in multicellular spheroids and in xenograft models.

## RESULTS

### New benzo[e]pyridoindolones

**Figure 1:**
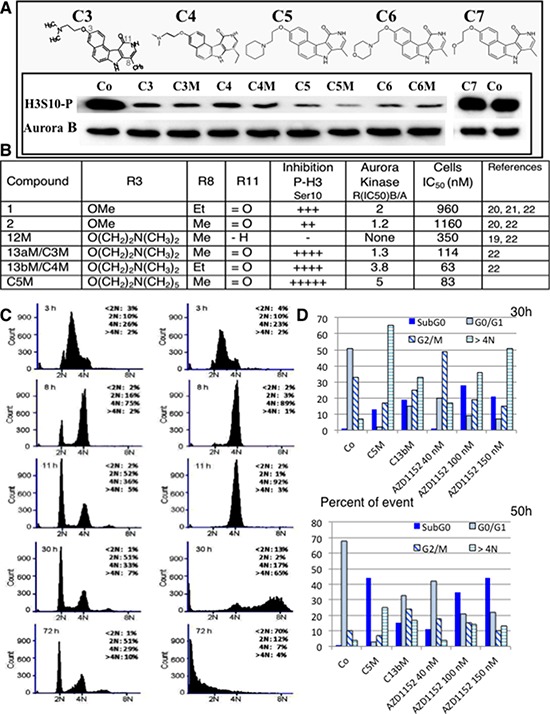
Efficiency of new benzopyridoindolones **A.** Inhibition of histone H3 phosphorylation. The molecules are represented on the upper part. Both C3 and C4 bear an amino-ethoxy group in position 3 and differ by the length of the alkyl group in position 8. C5, C6 and C7 respectively exhibit a pyperidine-ethoxy, a morpholino-ethoxy and a methoxy-ethoxy group. By condensation with maleic acid, we obtained the corresponding soluble salts (C3M to C6M). The inhibition activity was evaluated by western blotting. HeLa cells were incubated overnight with the different compounds (Cx and CxM, 500 nM) or DMSO (Co) in the presence of nocodazole (0.1 mg/mL) Blots were revealed by an anti-phospho Ser10-histone H3 (H3S10-P). Aurora B-kinase detection allowed the estimation of the amount of mitotic cells. **B.** The main information derived from our former work is recalled in a Table in comparison to C5M characteristics. **C.** Effect of C5M on cell cycle progression. HeLa cells were synchronized in S-phase by serum deprivation (48 h) and then, by thymidine block (20 h). Upon release, they were incubated with C5M (100 nM, shown on the right) for 3 h, 8 h, 11 h, 30 h and 72 h and compared to control (left part). DNA was stained with propidium iodine and samples were analysed by FACS. The percentage of cells in the different phases, except S-phase, was indicated in each panel. **D.** Comparative cell cycle progression in the presence of either C5M (100 nM) or C13b/C4M (nM) or AZD1152 (40 nM to 150 nM). Cells were synchronized as in part C and upon released they were incubated for 30 or 50 h in the presence of the drug then, cell cycle was conducted as above.

We previously showed that benzo[e]pyridoindolones (BePI) C3 and C4, recalled in Figure 1A and 1B, are potent inhibitors of aurora kinase B exhibiting antiproliferative activity in HeLa cells [22]. The presence of an amino function in the side chain opens the possibility to prepare the maleate salt of these BePI and to increase their solubility in water.

An Et substituent at position 8 was slightly more favourable than a methyl group, in terms of anti-proliferation activity (Figure 1B), but this slight gain of activity could not compensate the low yield of synthesis of these compounds. Actually, the first step is the preparation of an alkylated pyridone. Whereas the methylated pyridone derivative is prepared in 83% yield, with simple purification, the yield for the ethylated counterpart is not exceeding 25%, with a tedious purification. Therefore, the new compounds proposed are 8-Me analogues. It should be mentioned that the anchoring site for the amine chain was selected on the 3-position, as the importance of the substituent group at this position was demonstrated in our previous studies [22]. Indeed, the deletion of the methoxy group or their displacement from the 3- to the 4-position lead to less active compounds [22]. We therefore attempted to introduce side chains commonly encountered in medicinal chemistry like pyperidine-ethoxy, (C5) or morpholino-ethoxy chain (C6). These substitutions preserved and even increased the inhibitory activity estimated by the phosphorylation of histone H3 on Ser10 when compared to compound C3. Conversely the introduction of a methoxy-ethoxy group induced a loss of aurora kinase inhibition. C7 had similar inhibition than C2, a compound bearing an OMe at this 3-position [22], suggesting an advantage for the amine entity or at least for a large chain at this position. We confirmed, on this series, that the presence of the lactame group on BePI is a requirement for aurora kinase inhibition (Figure 1B, [22]). The free base compounds (Cx) and their corresponding maleate salt (CxM) had a similar aurora B-kinase inhibition. We decided to further characterized the most active compound 3-(2-(Piperidin-1-yl)ethoxy)-8-methyl-7*H*, 10*H*-benzo[e]pyrido(4, 3-b)indol-11-one maleate: C5M.

### Outcome of cells treated by benzo[e]pyridoindolones C5M

We tested the behaviour of cells treated with C5M. HeLa cells were synchronized at the S phase, and the compound (100 nM) was added upon release. Cell cycle analysis following 3, 8, 11, 30 and 72 h of compound treatment are presented in Figure 1C. After 11 h of treatment, cells in the control entered a new cycle (52% in G1) whereas the treated cells were primarily arrested in the G2/M population (92%). Finally these cells underwent polyploid, the > 4N population represented 65% after 30 h of incubation whereas none cells were cycling (only 2% in G1). Progressively, we observed the apparition of a sub G1 population of cells that accounted for 13% at 30 h and 70% at 72 h (Figure 1C). Meanwhile, C4M and AZD1152 induced mitotic arrest and polyploidisation but, some cells reinitiated a new cycle (Figure 1D), the proportion of the cells in G0/G1 was found high even after 50 h of treatment (33% for C4M and ranging from 41% to 22% for different AZD1152 concentrations). In conclusion, C5M differently affected mitotic cells compared to known drugs, it perturbed mitosis exit and normal cell cycling; it induced the polyploidisation and finally caused cell death.

### Antiproliferative activity of C5M

**Figure 2:**
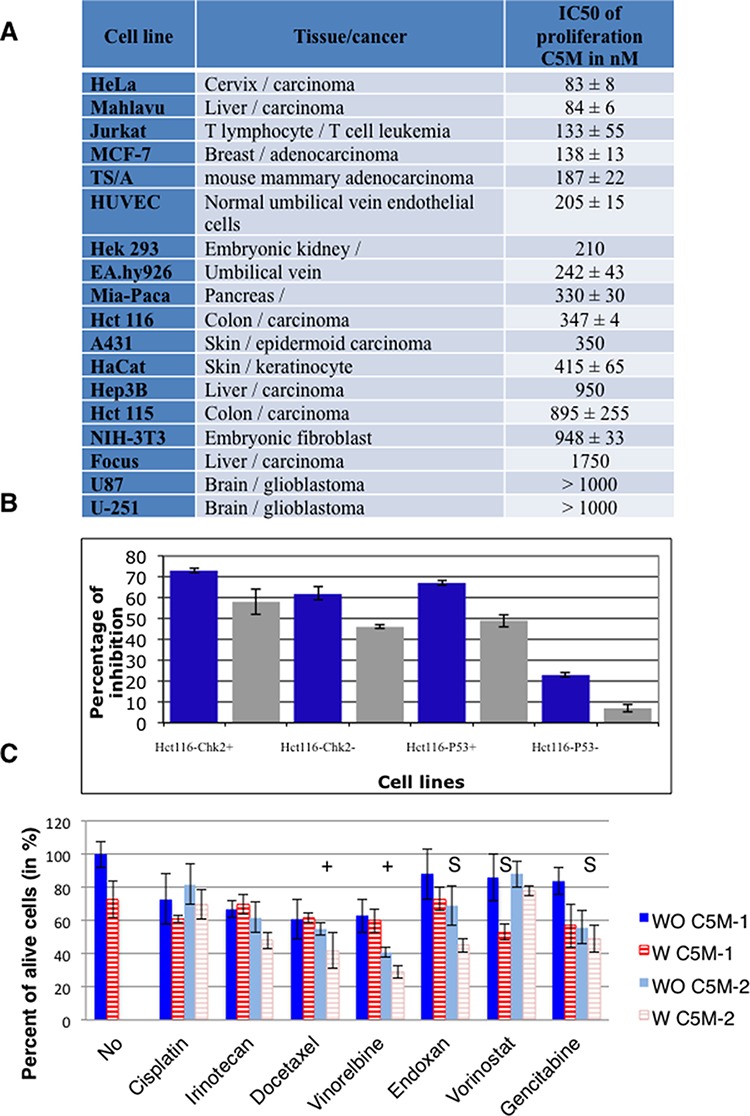
Antiproliferative activity of C5M **A.** the concentration of C5M reducing by half cell proliferation, after 96 h of treatment, was determined for a panel of cell lines. **B.** genetically modified hct116 cells were treated by two concentrations of C5M: 1 μM (dark histograms) and 250 nM (light histograms). **C.** different combined therapies were evaluated. The drugs were either added simultaneously (C5M-1) or C5M was first added when the second compound was supplied 24 hours later (C5M-2). These concentrations of the compound individually induced a decrease of viability of around 20%. The concentrations were as follows: C5M 50 nM, cisplatin 1 μg/mL, irinotecan 1 μM, docetaxel 1 nM, vinorelbine 0.4 nM, endoxan 0.5 μg/mL, vorinostat 400 nM and gemcitabine 2.5 μg/mL. (+) and (S) indicated that both treatments are respectively additive or synergic.

We evaluated the anti-proliferative activity of C5M through the viability of tumour HeLa cells upon 96 h of treatment. A concentration of 83 ± 8 nM was found to reduce by half HeLa cell proliferation (in-cell IC50). We extended this study to a large panel of cell lines (Figure 2A). We showed that C5M could efficiently prevent proliferation of cells from different origins like Mahlavu liver carcinoma (IC50 of 84 nM), Jurkat T-cell leukemia (IC50 of 133 nM), breast adenocarcinoma MCF-7 (IC50 of 138 nM). C5M efficiency varied a lot since high IC50 of several μM were also measured (Figure 2A). Interestingly C5M prevented normal HUVEC proliferation (IC50 of 205 nM). To start to understand these differences of efficiency we compared the C5M treatment in genetically modified hct116 cells. C5M appeared more efficient in p53^+^ than p53^−^ cells (at 1 μM, 65% of inhibition versus 22%) whereas Chk2 was not found important (Figure 2B). Only a slow decrease of efficiency was observed in its absence (at 1 μM, 61% of inhibition versus 72% in hct116 chk2^+^ cells).

We checked whether C5M could be combined to known therapies (Figure 2C). The association with DNA damaging agents (cisplatin and irinotecan) was not favourable. Alcaloid agents may have some benefit if the cells are primed with C5M since additive effects were observed. Vorinostat, an HDAC inhibitor, known to block cells in G2/M had a synergic effect if simultaneously added with C5M. C5M had a synergic effect with Endoxan, an inhibitor of transcription and gemcitabine, an analogue of deoxicitosine, when added upon one day of action.

### Time-lapse experiments

**Figure 3:**
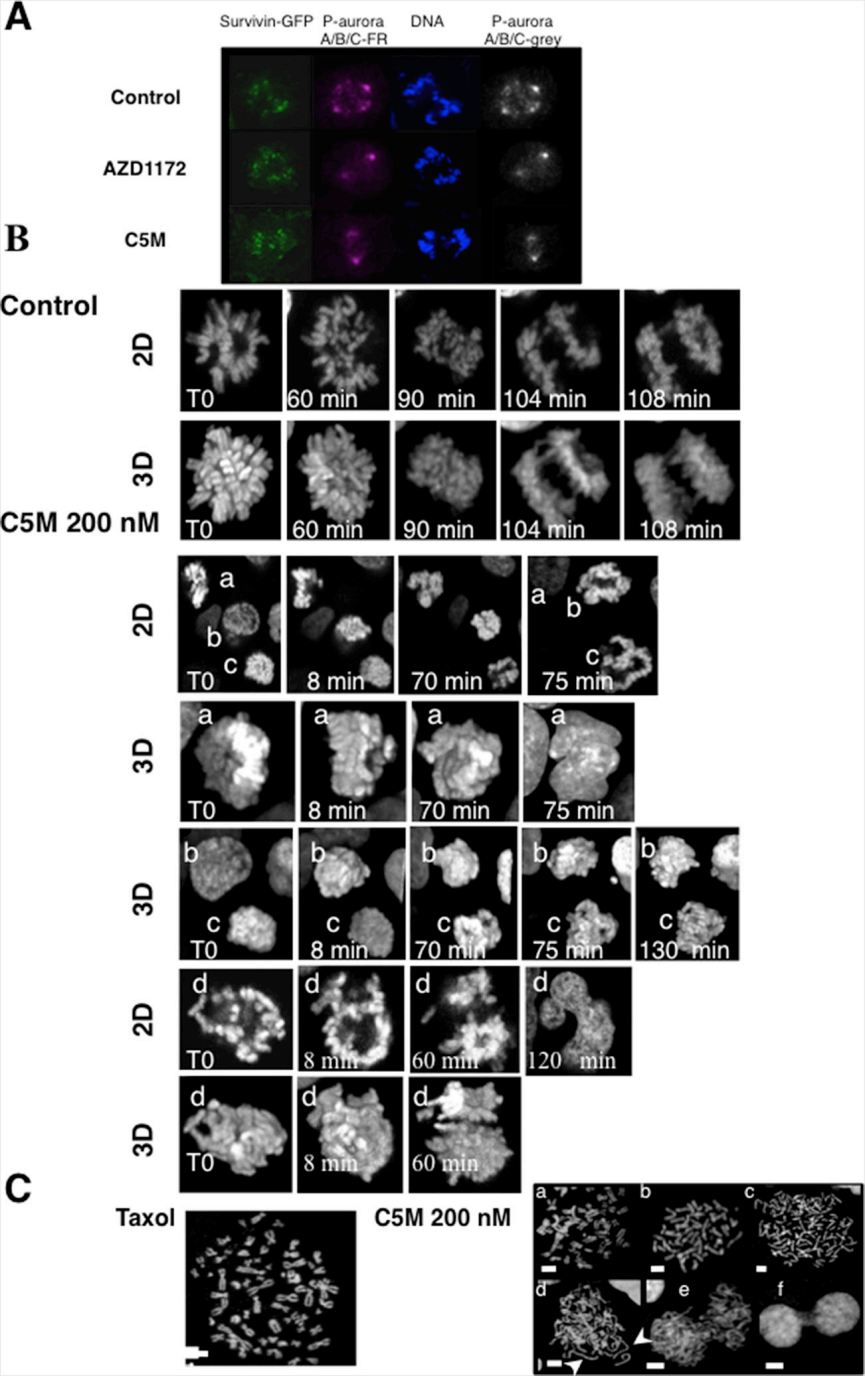
Visualization of mitotic chromosomes in the presence of C5M In part **A.** phospho-aurora A/B/C were detected with a specific antibody. S12 cells expressing survivin-GFP were either treated for 20 h by AZD1152 (200 nM) or C5M (200 nM) and compared to control cells. Phospho-Aurora A/B/C was stained with far-red antibodies and the staining was turned to grey on the right part while DNA was labelled with Hoechst. In part **B.** a time-lapse of mitotic Hek-293 cells stably expressing Histone H2A-GFP is presented. Z-stacks were acquired and both 2D-images and 3D-projections are shown in each experiment. Elapse times are indicated on each photo. In the control a single mitotic cell is followed whereas four different cells (labelled a to d) illustrated the effect of C5M (200 nM). In part **C.** spread metaphases were imaged. The effects of C5M (overnight, 200 nM) illustrated by several representative situations are compared to taxol. DNA was stained by Hoechst. Long decondensed sister chromatids are indicated by arrows.

First, we took advantage of a specific phospho-aurora antibody that recognized activated kinases A/B and C to check the specificity of C5M, within this family, in HeLa cells (Figure 3A). Centromeres were decorated by survivin-GFP. C5M and AZD1152 prevented the phosphorylation of aurora B (Thr232) on the centromere whereas the centrosomes were still decorated by phospho-aurora A (Thr288). In cells, C5M (200 nM) targeted Aurora B not A.

To better describe the action of C5M we performed time-lapse experiments in Hek-293 cells stably expressing histone H2A-GFP. Mitotic cells were selected and continuously imaged. C5M was added just before imaging and the treated cells were compared to control (Figure 3B). In control, the prometaphasic cell underwent in metaphase (*t* = 60 min), then in anaphase (90 min) and finally the two lots of chromosomes segregated (104 min). In the presence of C5M, four different mitotic cells are shown (from a to d), a 2D-plan and a 3D-reconstitution are provided. At T0, a appeared as a metaphasic cell but, 70 min later, chromosomes are still compacted and finally, at 75 min, a became a binuclei interphasic cell as clearly visualized on the 3D image. Cell b was in prophase at T0 and progressed in mitosis but chromosome segregation failed. Cell c was in prometaphase at T0 and went in metaphase (8 min) and chromosomes partly segregated but they were still connected at 130 min. Cell d also illustrated the failure of chromosome segregation or at least a non-equilibrated separation that conducted again to a polynuclei cell (Figure 3B). We observed HeLa spread metaphases (Figure 3C). Cells were enriched in mitosis either by taxol (33 nM) or by C5M (200 nM). In the taxol experiment, we noted resolved chromosomes connected by their centromere. The situation is more complex in cells treated by C5M since in addition to normal metaphasic figures (a and b), we had a large proportion of cells exhibiting individual chromosomes (c and d). In d, individual chromosomes seemed to be decondensed, a feature not expected upon anaphase onset since maximal compaction occurs in late anaphase. The proportion of individual chromatid versus attached chromatid is around 73% with C5M for less than 2% with taxol. We could also note the presence of cells with mis-segregated chromosomes or connected chromatin (e and f) that were never observed with taxol.

**Figure 4:**
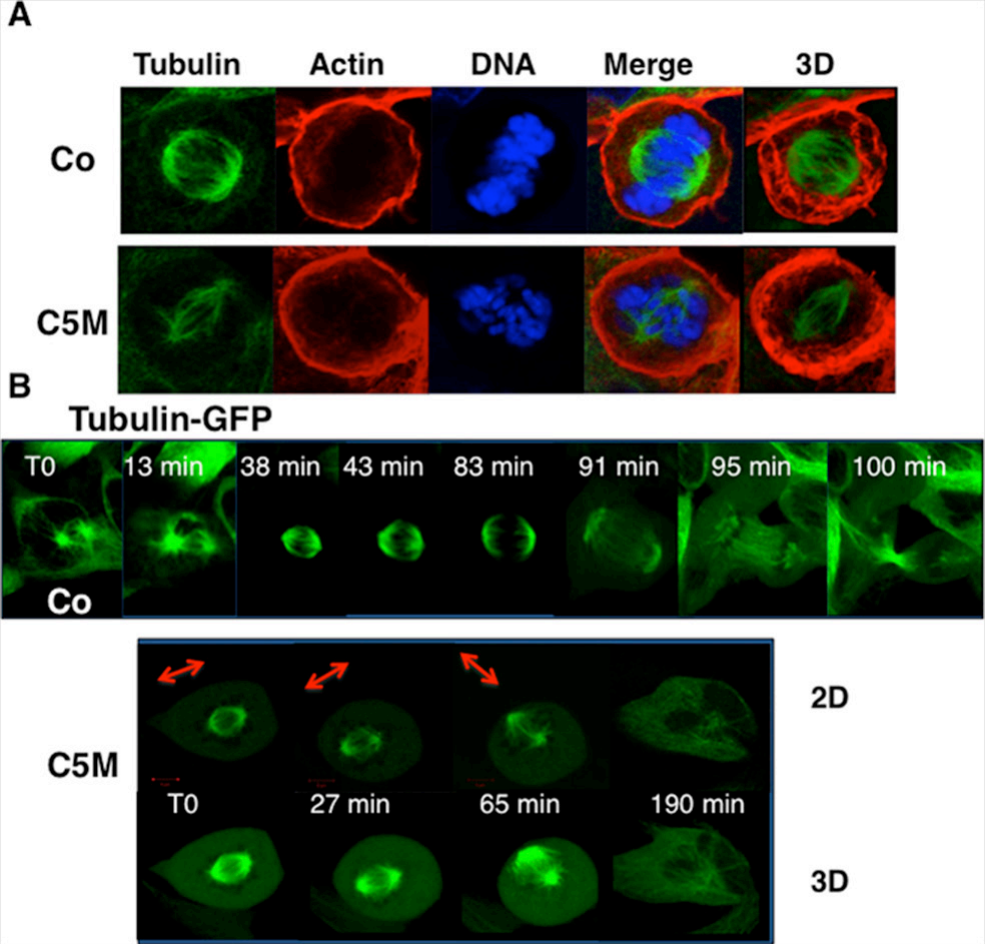
Perturbation of the mitotic spindle by C5M In part **A.** immunofluorescence of HeLa cells under control conditions (Co) or upon C5M treatment (ON, 100 nM). Tubulin, actin and DNA were simultaneously detected. A Z-slice and a 3D-projection (tubulin and actin) are shown. In part **B.** a time-lapse experiment was realized under control conditions (Co) or upon C5M treatment (200 nM) with Hek-293 cells expressing GFP-α-tubulin. Elapse times are indicated on each image. A 3D-projection is shown for C5M treated cells to better visualize the spindle orientation that is also illustrated with an arrow.

In mitosis, the axis of division is driven by cytoskeleton and is pre-established before metaphase. Under C5M treatment, we observed unusual movements of chromatin (Figure 3B, cells c t70 and 75 min and cell d T0 and 8 min) and we wondered what happened to the mitotic spindle (Figure 4). Immunofluorescence experiments realized on HeLa cells revealed that the tubulin spindle was reduced in the presence of C5M and did not seem to be stabilized by the actin basket as in the control (compare the 3D-projections in Figure 4A). Time-Lapse experiments with Hek-293 cells expressing GFP-α-tubulin allowed the description of the mitotic spindle under C5M treatment (Figure 4B). In control conditions, the two centrosomes appeared, they migrated at opposite sites and they indicated the orientation of the spindle. In metaphase, the spindle is at equal distance from the cell surface (Figure 4A). Upon C5M treatment, the spindle was found normally localized at mitotic entry but then, it loose this central localization and finally turned in the opposite direction (Figure 4B, T0, 27 min and 65 min). Progressively the spindle disappeared and the cell spread on the substrate, escaping mitosis without any segregation (neither chromosomes, nor cytoplasm, Figure 4B, 190 min). The mis-orientation of the spindle and its regression was systematically observed in the presence of C5M.

### C5M prevented AMPK-Thr172 phosphorylation in late mitosis

**Figure 5:**
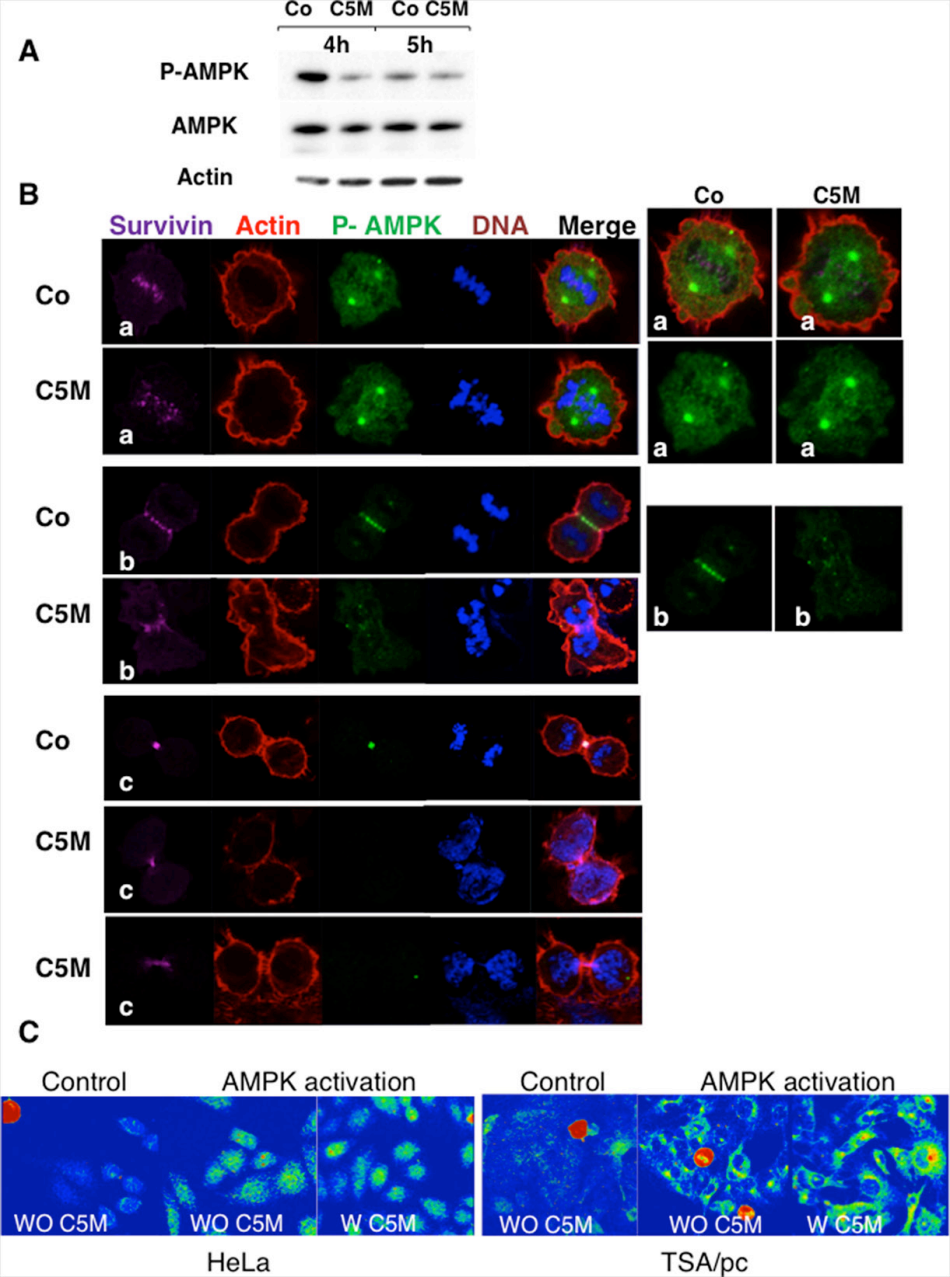
Detection of phosho-Thr172-AMPK in mitosis **A.** mitotic cell extracts were analysed by immunoblotting. Cells treated by C5M or under control conditions were recovered 4 and 5 hours post release from nocodazole. AMPK and phospho AMPK were detected as well as actin (loading control). **B.** immunofluorescence were realized on HeLa cells treated by C5M (100 nM, ON) and compared to control conditions (Co). A metaphase, an anaphase and telophases are shown. Survivin is in far-red, actin in red, phospho-Thr172-AMPK in green and DNA in blue. Enlarged views of phospho-AMPK are proposed on the right part. **C.** detection of phospho-Thr172-AMPK on HeLa or TSA/pc cells stressed by high salt treatment for 10 min at 37°C. Cells pre-incubated with C5M (W C5M 200 nM) for 1 hour before stress induction ([43]) were compared to untreated stressed cells (WO C5M). The signal was turned to a rainbow scale: blue for negative, green to yellow for positive signals and red for the highest signals. Mitotic cells are in red whatever the treatment.

In the literature, phospho-AMPK was described as a new mitotic mark that could play a role in spindle stabilization/orientation [23] [24]. We decided to check whether C5M impairs AMPK phosphorylation in mitosis (Figure 5A). We synchronized cells by nocodazole and then released them with and without C5M (Co). Mitotic cells were recovered by flushing and proteins were extracted. After 4 h of release in the presence of C5M (100 nM), we observed an important decrease of phospho-AMP-Thr172 compared to the control (Figure 5A). By immunofluorescence, on metaphases, we found that phospho-AMPK is present in the centrosomes whatever the treatment but, mild differences appeared on centromeres (Figure 5B, a). In control conditions, centromeres decorated by survivin were surrounded by phospho-AMPK suggesting an outer centromeric localization for this mark. Conversely phospho-AMPK signal was more diffuse in metaphasic C5M-treated cells (Figure 5B, a). In anaphase, phospho-AMP-Thr172 was found mostly present on the spindle, in close contact to survivin (Figure 5B, b). Upon C5M treatment survivin was transferred to the spindle but the phospho-AMP-Thr172 signal was very low and poorly organized (Figure 5B, b). Finally on telophase, phospho-AMP-Thr172 and survivin co-localised on the mid-body (Figure 5B, c). C5M perturbed the mid-body organization and clearly the survivin and phospho-AMP-Thr172 signals were not co-localised (Figure 5B, c). Actually, except in pro-metaphases, the phospho-AMP-Thr172 signal was very low in C5M treated cells (Figure 5B). The next question was the possible inhibition of AMPK in interphasic cells. We noted that the acetylation of histones H3 and H4 did not decreased, in cells, whatever the time of treatment (1 h, 26 h or 48 h, data not shown) indicating no long term impairment of this signalling [25]. The possibility of a short-term inhibition was evaluated. We activated AMPK in HeLa and TSA-pc cells by a hypertonic stress for 10 min. As shown in Figure 5C, the signal of phospho-AMPK (Thr172) was found identical in control (WO C5M) and in cells pre-incubated with C5M (200 nM, for 1 h, W C5M). The efficiency of C5M upon one hour of treatment was verified on anaphase cells.

**Figure 6:**
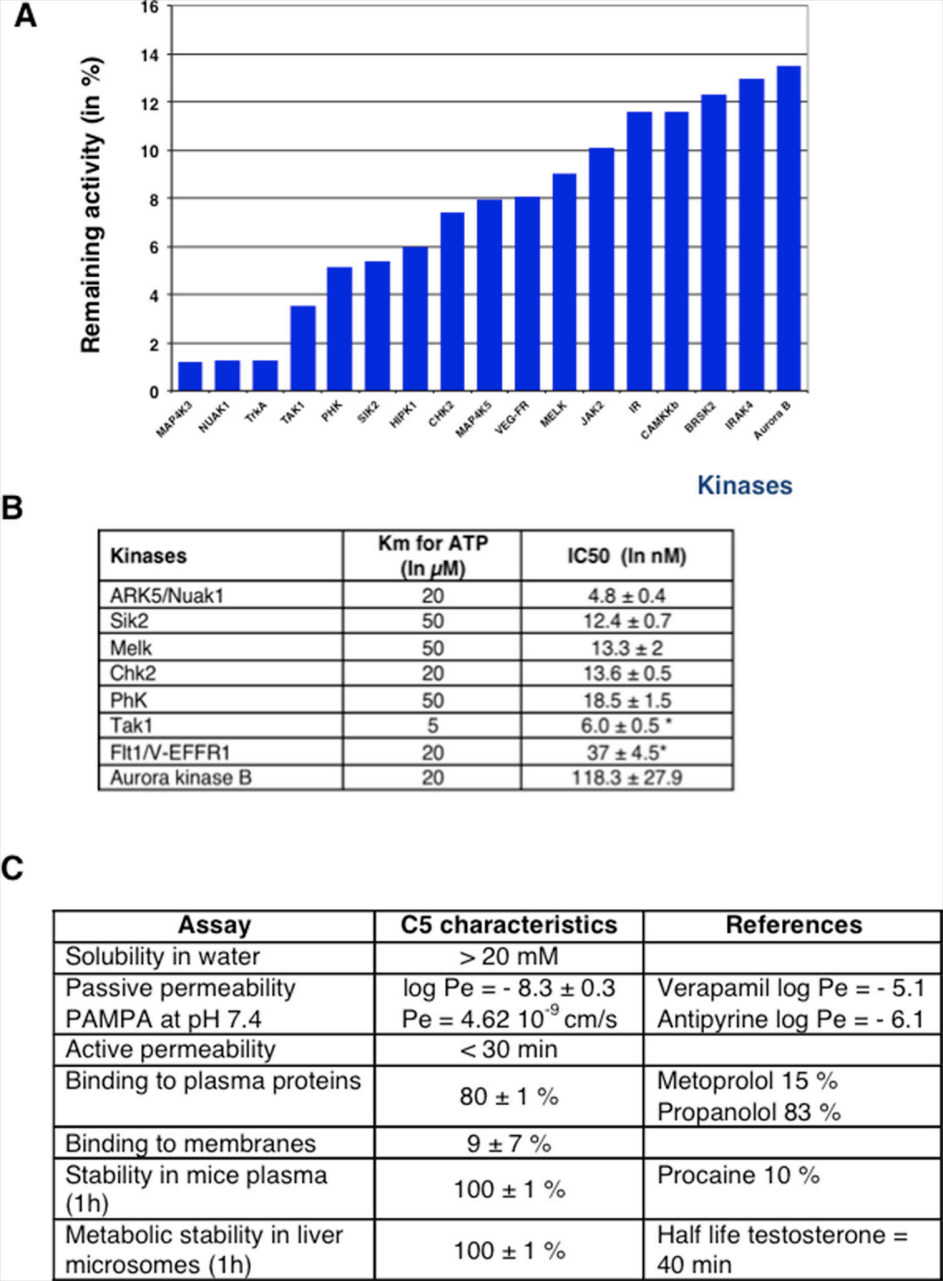
*In vitro* characterization of C5 A profiling was realized against 138 kinases at the concentration of 250 nM. The best inhibitions are highlighted by the histograms **A.** IC50 determinations are reported in part **B.** Stars indicated that the IC50 were determined with an ATP concentration of 1 μM instead of the Km concentration. Pharmacokinetic characteristics are reported in **C.** and compared to approved drugs (indicated as references).

The extinction of phospho-AMPK signal in late mitosis prompts us to characterize the profile of inhibition of C5M (250 nM) towards 137 major kinases. C5M was found to be a multikinase inhibitor with restraint selectivity for few kinases (Figure 6A). C5M is specific to aurora B-kinase versus aurora-A and we determined an IC50 of 118 nM for kinase B (Figure 6B). Among the fifteen best targets, we noted the presence of several AMPK-related kinases (Nuak1, Sik2 and Melk) as well as AMPK upstream regulator kinases (Tak1 and CaMKKβ). These data were confirmed by the determinations of few *in vitro*-IC50 (Figure 6B). *In vitro*, IC50 found for the AMPK–related kinases ARK5/Nuak1, Sik2, Melk and Tak 1, are 4.8 nM, 12.4 nM, 13.3 nM and 6 nM respectively. Interestingly we reported IC50 of 13.6 nM for Chk2 and Chk1, 18.5 nM for phosphorylase kinase and 37 nM for Flt1/V-EGFR1. To further evaluate C5M, some preclinical characteristics were determined (Figure 6C). C5M was found to be hydrosoluble with solubility higher than 20 mg/mL in water. Its passive permeability across reconstituted membranes was found low but time-lapse experiments indicated a quick cytoplasmic distribution upon addition on cells. C5M had quite no affinity for membranes (9% retained) and bound to plasmatic proteins (80%). This molecule had a nice stability in plasma as well in liver microsomes since 100% of C5M was recovered after 1 h of incubation (Figure 6C).

### Anti-proliferative activity in spheroids and *in vivo*

**Figure 7:**
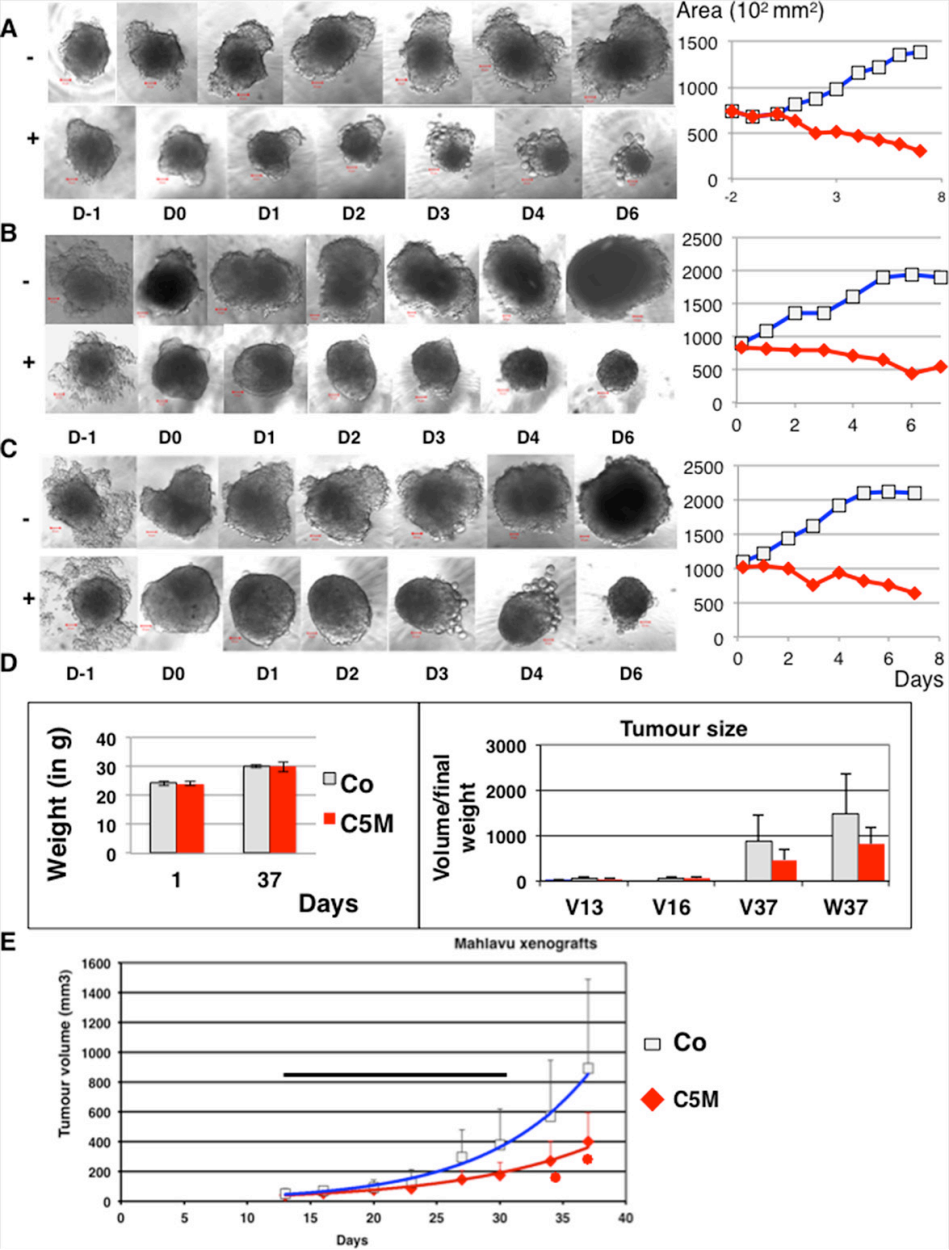
Effect of C5M on mixed spheroids and *in vivo* In **A.** TS/A-pc spheroids were incubated with (+) or without (−) C5M (1 μM) and followed for 7 days post drug addition. A representative spheroid is shown in each situation before drug addition: D-1 and D0 and upon 1, 2, 3, 4 and 6 days of treatment. The bar represents 50 μm. The mean area of the larger section of spheroids was plotted as a function of the time of treatment, square for the control and diamond for the treatment. Mean of the surface of the five spheroids are: 137 960 ± 34 060 μm^2^ for the control and 30 000 ± 5 340 μm^2^ for treated spheroids. The measures of the height are less precise but fit with these variations. In **B.** the same experiment as in A except that the spheroids were composed of TS/A-pc and normal fibroblasts added at Day 0. Mean of the surface of the five spheroids are: 191 260 ± 49 180 μm^2^ for the control and 56 100 ± 21 490 μm^2^ for treated spheroids. In **C.** the same experiment as in A except that the spheroids were composed of TS/A-pc and normal fibroblasts and Ea.Hy926 endothelial cells, both added at Day 0. Mean of the surface of the five spheroids are: 210 074 ± 48 030 μm^2^ for the control and 64 320 ± 9 060 μm^2^ for treated spheroids. In **D.** and **E.** Malhavu xenografts were established in nude mice and then animals were treated subcutaneously 11 fold from day 13 to 31. Mice received 5 mg of C5M per kg per injection. In D, the left histograms represented the weight of the mice. The mean volumes of the tumours (V in mm^3^) as well as their final weights (W) are reported in the right part. Light bars: mice receiving the vehicle and solid bars: mice treated by C5M. In E, the evolution of the tumour volumes over time are represented, square for control and diamond for treated animals. The horizontal bar figures the treatment period and stars indicated significant differences (student test *P* ≤ 0.05).

Altogether these data indicated that C5M could have potency as antiproliferating drug. We therefore decided to evaluate C5M on tumour models. First, we incubated C5M (1 μM) with complex spheroids composed of mammary tumour cells (TS/A-pc) alone or mixed with normal fibroblast (NIH-3T3 cells) and endothelial cells (Ea. hy926 cells) in order to mimic tumour heterogeneity (Figure 7A–7C). Spheroids were around 300 μm in diameter when the drug was added and they were followed for 7 days. Control spheroids grew exponentially as indicated by the curves ((Figure 7A–7C, diamond symbols). At day 2, we already observed an impressive decrease of the TS/A-pc spheroid size (a decrease by 43% of the area encompassing the largest ”diameter”, see Figure 7A, square symbol). Finally, large cells appeared in the periphery and the treated spheroids were quite five times smaller than in control. A single dose of C5M prevented the growth of mixed spheroids and even decreased their size by 74% and 70% for TS/A-3T3 and TS/A-3T3-Ea. hy respectively within 7 days of treatment (Figure 7B and 7C).

Moreover spheroids were found to remodel during the experiment whereas in the presence of C5M they mostly keep a round, compact shape (Figure 7B and 7C especially). In conclusion, C5M was found efficient towards a 3D-structure mimicking the complex tumour environment and its effect lasted through time. We wonder whether C5M could be safely injected to animals. We established Malhavu xenografts in nude mice and repeatedly injected subcutaneously animals with either C5M or the vehicle. The autopsy of the animals performed by a veterinarian revealed no macroscopic adverse effects (data not shown) and the animals did not lose weight (Figure 7D). Animals were sacrificed 6 days after the last injection. The tumours were significantly smaller in the treated group compared to control (Figure 7D and 7E). C5M subcutaneously injected was found not toxic for the animals and could prevent xenograft development. Again its effects lasted through time.

## DISCUSSION

Benzo[e]pyridoindolone bearing a pyperidine-ethoxy chain, named C5, is a potent aurora B kinase inhibitor. The synthesis is realized in 10 steps ending by the maleic salt formation to obtain a hydrosoluble compound (C5M). The molecule is rather small (MW 377 for the free base), a characteristic reported to be positive for clinical trials.

Cell cycle analysis of HeLa cells treated by C5M (100 nM) indicated a strong mitotic arrest then, cells became polyploid and finally died. C5M is a high antiproliferative mitotic drug. C5M could efficiently prevent proliferation of cells from different origins like Mahlavu liver carcinoma, Jurkat T-cell leukemia, MCF-7 and TS/A-pc breast adenocarcinoma, but also endothelial proliferative HUVEC.

Fluorescent time-lapse experiments revealed how C5M affected mitosis on-going. The effects were observed without delay suggesting good cell permeability. Chromosomes condensed and quite perfectly aligned in metaphase. But even when two lots of chromosomes could be discriminated they did not segregate. Actually, the daughter chromatids were found separated, neither the cohesin removal, nor the centromere separations were perturbed. The full condensation arising in late anaphase seemed affected since we observed long individual chromatids. During anaphase, a maximal chromosome compaction was described that occurred by a mechanism of axial shortening of the chromatid arms from telomere to centromere and whose impairment resulted in multilobed daughter nuclei [26]. This chromatid axial shortening was reported to depend on dynamic microtubules and aurora B kinase activity [26]. Most aurora B kinase inhibitors prevented chromosome alignment on the metaphasic plate, induced a delocalisation of aurora kinase from centromere on the whole chromosome and finally the cells escape from mitosis [19]. Although C5M prevented histone H3 phosphorylation, it mostly affected late mitosis steps since aurora kinase and survivin were transferred to the central spindle, a sign of anaphase onset. The transfer of the Chromosomal Passenger Complex (CPC) on the mitotic spindle was not reproduced with AZD1152, a specific aurora B kinase inhibitor [27]. These peculiar features of C5M could be due to the simultaneous inhibition of mitotic off-targets like Chk1 or Melk (*in vitro* IC50 around 13 nM) in addition to aurora B. Melk belongs to the AMPKr family and was mostly involved in cytokinesis [28]. Checkpoint kinase 1 (Chk1) is a well-established component in the DNA damage and DNA replication pathways [29] but it is also involved in optimal chromosome segregation as well as in spindle checkpoint signalling. Chk1 is required for correction of merotelic attachments during a prolonged metaphase and it phosphorylates aurora-B at Ser331 a phosphorylation essential for optimal spindle checkpoint function [30, 31]. C5M effects could thus result from the co-inhibition of several mitotic kinases. C5M, with its unusual aurora kinase inhibition profile, is a tool for proving aurora B actions in late mitosis. Sharp specific aurora B inhibitors were described to induce neutropenia [32], an adverse side effect that could limit their clinical use. In contrast, the potential benefit/risk window provided by drugs with the multi-targeting strategies still need to be addressed but may present lower toxicities. Presently several multikinase inhibitors are in clinical trials [33].

During this study, we could not detect phospho-Thr172 AMPK on the mitotic spindle whereas this phosphorylation was present on centrosomes. AMP-activated protein kinase (AMPK) firstly studied as a central metabolic stress sensor was described to ensure proper cell division and faithful chromosomal segregation during mitosis. P-Thr172 –AMPK corresponded to a fully activated kinase but the modification was found dispensable for maximal activity [34]. AMPK could be phosphorylated by LKB1, calmodulin-KKb and TGF-Δ-activated-k1 [35]. Our observations were performed in LKB1 negative cells, but CAMKKb and Tak-1 are *in vitro*-targets for C5M, appearing in the top fifteen. Moreover it was reported that AMPK regulates mitotic spindle orientation through the phosphorylation of myosin regulatory light chain (P-MLRC) [24]. C5M perturbed spindle orientation but MRLC was found fully phosphorylated (data not shown). The spindle defects preceded the decrease of AMPK phosphorylation that could be due to the absence of stabilisation/tension on centrosomes. Does C5M directly prevent AMPK phosphorylation or is it a consequence of an inactive CPC, the question is opened. Actually, AMPK substrate like acetyl-CoA carboxylase [36] and phospho-active forms of proteins belonging to the AMPK/mTOR/S6K1 signalling axis localized on the cleavage furrow in close vicinity to the CPC [37]. How AMPKα activation is controlled spatially and temporally during mitosis, remained undiscovered. C5M could be useful for understanding the role of phospho-AMPK, in abscission and the relationship between the CPC and the energy sensing machinery. We noted that C5M had no effect on interphasic and centrosomic phospho-AMPK signals. The mitotic selectivity of C5M and its failure of inhibition of metabolic AMPK could be positive features in terms of cancer therapy. Actually, several clinical assays are based on AMPK activation by metformine [38]

C5M is an efficient mitotic anti-proliferative drug that could target both tumour cells and neo-vessels around. C5M prevented the proliferation of endothelial cells, possibly through Flt-1/V-EGF-R1. The preclinical characterization revealed its high hydro-solubility and a good stability in plasma as well in liver microsomes. Accordingly, a single dose was found efficient for preventing tumour growth in multicellular spheroids and quite long-term effects were noted in liver Malhavu xenografts in nude mice. *In vivo* assays demonstrated that C5M could be safely injected to mice. The next step will be to define the mode and area of applications of this new anti-proliferative drug and to evaluate its potency as a radio-sensitizer, a property of the parental molecule C1 [21]. Combined chemotherapies including C5M could be promising.

## MATERIALS AND METHODS

Thymidine, nocodazole, phalloidin-rhodamin, propidium iodide and AZD-1152 were from Sigma–Aldrich. RNase A was supplied by Euromedex.

### Synthesis

Benzo[e]pyridoindole synthesis and characterization were partly described [20, 39, 40].

Synthesis of C5 and C5M are synthesized as follows:

*Step 1*: 11-Chloro-3-(2-(piperidin-1-yl)ethoxy)-8-methyl-7*H*-benzo(e)pyrido(4, 3-b)indole

**Figure:**
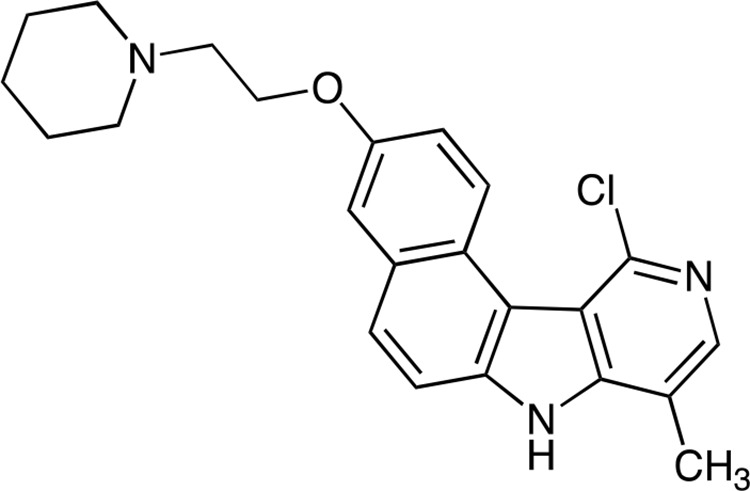


A mixture of 11-chloro-3-hydroxy-8-methyl-7*H*-benzo(e)pyrido(4, 3-b)indole prepared as described in [41] (1.00 g, 3.5 mmol), *n*-butanol (60 mL) and water (40 mL) was stirred for 30 min and then a solution of NaOH (900 mg) in water (10 mL) was added. Stirring was continued for an additional 15 min then N-(2-chloroethyl)piperidine hydrochloride (790 mg, 4.2 mmol) was added and the mixture was heated under reflux for 2.5 h. The reaction mixture was cooled and organic layer was separated. The aqueous layer was extracted by AcOEt and the organic layers were combined, washed with brine, dried over MgSO_4_ and evaporated in vacuum. The residue was purified by flash chromatography (neutral alumina, gradient of ethanol (0 to 4%) in dichloromethane) to give the expected compound as beige solid (1.20 g, 72%). ^1^H NMR (300 MHz, DMSO-d_6_) δ (ppm): 12.48 (br s, 1H), 9.61 (d, 1H), 8.06 (s, 1H), 7.99 (d 1H), 7.79 (d, 1H), 7.58 (d, 1H), 7.34 (dd, 1H), 4.22 (t, 2H), 2.74 (t, 2H), 2.56 (s, 3H), 2.51–2.45 (m. overlapped by DMSO signals), 1.56–1.47 (m, 4H), 1.45–1.30 (m, 2H). Microanalyses, calculated for C_23_H_24_ClN_3_O_2_: C, 70.13; H, 6.14; N, 10.67, found: C, 69.63; H, 6.38; N, 10.44. MS 394.1 & 396.1 [M+1].

*Step 2*: 3-(2-(Piperidin-1-yl)ethoxy)-8-methyl-7*H*, 10*H*-benzo(e)pyrido(4, 3-b)indol-11-one maleate (C5)

**Figure:**
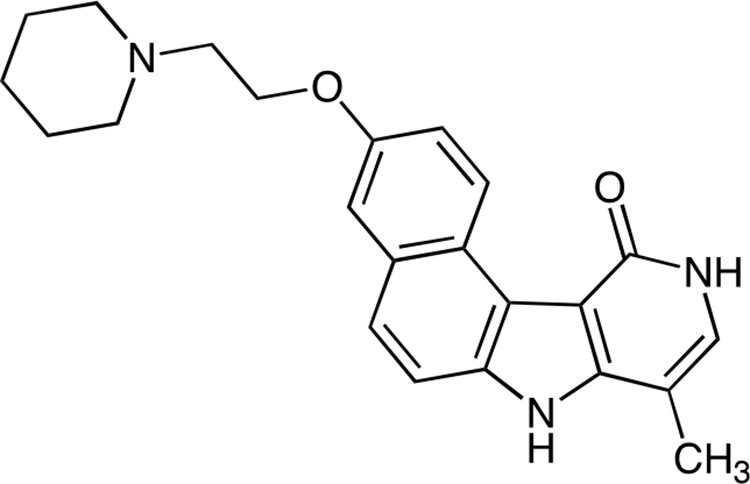


A mixture of 11-chloro-3-(2-(piperidin-1-yl)ethoxy)-8-methyl-7*H*-benzo(e)pyrido(4, 3-b)indole (630 mg, 1.60 mmol), sodium acetate (600 mg, 7.2 mmol) and AcOH (40 ml) was heated under reflux, for 18 h. The volatile material was first evaporated in vacuum and then co-evaporated with toluene. Water (150 ml) was added and the medium was basified by addition of sodium hydroxide (2 N). The aqueous layer was extracted by AcOEt/EtOH:95/5, dried over MgSO_4_ and evaporated in vacuum to give the title free base of C5 (590 mg, 98%).^1^H NMR (300 MHz, DMSO-d_6_) δ (ppm): 12.03 (br s, 1H), 10.94 & 10.92 (2 br s, 1H), 10.26 (d, 1H), 7.73–7.63 (m, 2H), 7.41 (d, 1H), 7.17 (dd, 1H), 7.08 (d, 1H), 4.18 (t, 2H), 2.73 (t, 2H), 2.56–2.43 (m, overlapped by DMSO signals), 2.29 (s, 3H), 1.57–1.48 (m, 4H), 1.44–1.35 (m, 2H). Microanalyses, calculated for C_23_H_25_N_3_O_2_.H_2_O: C, 70.22; H, 6.87; N, 10.68, found: C, 70.16; H, 6.92; N, 10.42. MS 376.2 [M+1].

Formation of the maleate salt: A solution of this free base (300 mg) in boiling absolute ethanol (15 mL) was poured into a solution of maleic acid (100 mg) in hot absolute ethanol (4 mL). The homogenous solution obtained was evaporated under vacuum and the residue was triturated with acetone giving a solid which was collected by filtration, washed with acetone and dried in a desiccator affording the maleate salt of C5 (370 mg). Microanalyses, calculated for C_23_H_25_N_3_O_2_.C_4_H_4_O_4_.0.5 H_2_O: C, 64.80; H, 6.00; found: C, 64.41; H, 6.37.

### Compound characterization

The half maximal inhibitory concentration of the compound (IC50) and the kinase profiling were performed at the MRC (Dundee) with a panel of recombinant enzymes. All assays were run in duplicate and the ATP concentration adjusted at the Km value for each kinase. Preclinical determinations were done at the CNRS-platform TechMed^ILL^ (Strasbourg, France; http://www.pcbis.fr/fr/TechMed-IntroductionPresentation)

### Cell lines

Most cell lines obtained from ATCC were cultured under suggested conditions. HUVEC cells from Lonza (Levallois, France) were cultured as suggested by the supplier. TS/A-pc cells were cultured in RPMI 1640 media supplemented with 1% glutamine, 10% foetal bovine serum, 25 μM β-mercaptoethanol. HeLa cells stably expressing survivin-GFP and Hek-293 expressing either histone H2A-GFP or GFP-α-tubulin were already described [20, 42]. Genetically modified hct116 cells were a gift from Dr B. Vogelstein. HeLa cells were synchronized at S phase by serum deprivation for 2 days followed by a thymidine block (3 mM) for 20 hours. After PBS washing, cells were then either allowed to grow in normal condition or in the presence of C5M (100 nM). Alternatively, HeLa cells were synchronized in prometaphase by nocodazole (0.33 μM, ON). For stress induction, culture medium (or PBS for H_2_O_2_) was replaced by freshly prepared medium to which 125 mM NaCl was added, yielding a final concentration of 275 mM [43]. Compounds for combined chemotherapy were from Sigma Aldrich.

### Multicellular tumour spheroid (MTS) models

Spheroids were first generated by plating TS/A-pc cells at 2000 cells/well into ultra low adherence -96-well plates (Corning, Tewksbury, USA) [6]. These plates stimulate spontaneous formation of a single spheroid of cells, within 24 hours of incubation at 37°C, 5% CO_2_. Three day later, medium or 1000 NIH-3T3 cells or 800 NIH-3T3 and 800 EA. hy926 cells were added to wells. Next day (D0 on Figure 7) medium or C5M (final concentration of 1 μM) were added. The final volume is the same for all the wells (200 μL/well). Spheroids culture is performed in RPMI 1640 media supplemented with 1% glutamine and 10% foetal bovine serum.

Spheroids were imaged, each day, with a 10-X objective and then the area of the larger section was measured for each spheroid by Image J software.

### Western blot

Cells treated by compounds or DMSO (control, Co) or PBS (Co) were harvested and lysed in 9M urea, finally supplemented with Laemmli sample buffer as described previously [40]. AMPK detections were performed on RIPA-cell extracts. Western blots were performed using the following antibodies: rabbit anti-phospho-histone H3 (Ser10) (Upstate, 1:2000); rabbit anti-aurora B (Epitomics, 1:5000); rabbit anti-phospho-AMPK (Thr172) (Cell Signaling, 1:1000); rabbit anti-AMPK (Cell Signaling, 1:1000); mouse Δ-actin (Sigma, 1:5000). Bands were visualized by horseradish peroxidase labelled antibodies and ECL technique (Amersham Bioscience). Images were observed by ChemiDoc MP system (Biorad).

### Cell cycle analysis

HeLa cells were synchronized at S phase. After release, they were treated by C5M (100 nM), for 3, 8, 11, 30 and 72 hours and compared to control cells. For determination of cell cycle profiles, cells were fixed by ice-cold 70% ethanol, for 1 hour, and then, incubated with propidium iodide solution (50 μg/ml) in the presence of 0.2 mg/ml RNase A for 15 minutes, at 37°C. DNA content was measured using the BD Accuri C6 flow cytometer (BD Biosciences, US) and CFlow Plus software.

### Cell viability

Cell proliferation assays were conducted in 96-well culture plates. Assays were run in triplicate. Serial dilutions of compounds were prepared from 10 nM to 2 μM and the viable cell number was determined, at day 4, by the addition of MTS (Promega) or WST-8 (Promokine). In most cases, two to three different experiments were run.

### Immunofluorescence

Hela cells grown on glass coverslips were treated by C5M (100 nM) or water (control, Co) in different trials and then, fixed by formaldehyde 4% for 10 minutes at 37°C. Immunofluorescences were performed as described previously [40]. Coverslips were incubated with the antibodies directed against the following antigens: phospho-histone H3-Serine10 (Upstate, 1:2000); phospho-histone H3-Serine 28 (Upstate, 1:2000); aurora kinase B (Epitomics, 1:2000); rabbit anti-phospho-AMPK (Thr172) (Cell Signaling, 1:100), rabbit anti-phospho-aurora A/B/C (Cell Signaling, 1:100). Actin was stained by phalloidin-rhodamin (1 μg/ml). DNA was visualized with Hoechst 33342 (Sigma, 0.5 μg/ml). Images were collected with a ZEISS 710 Laser Scanning Confocal microscope with a 63×-immersion oil objective. Slices of 0.5 μm are shown.

Metaphases spreading were realized as follows: HeLa cells were incubated ON with either C5M (200 nM) or taxol (33 nM) and were then recovered by flushing. An osmotic choc was realized (calf serum diluted in water 1:5) for 13 min, at 37°C, then the same volume of fixative (mixture of EtOH and acetic acid (3:1)) was added. After 20 min of incubation at room temperature, cells were washed by fixative and stored at 4°C. Finally cells were spread on cold coverglass and DNA was stained with Hoechst.

### Time lapse

*Ex vivo* experiments were conducted on cells grown on Lab-Tek chambered coverglass (Nalgen Nunc International) and maintained under standard culture conditions (37°C, 5% CO_2_). Images were acquired on a Zeiss dynascope confocal microscope using a PlanApochromat 40× water immersion objective. Images were analysed with the Zen software provided by Zeiss. C5M was added just before imaging.

At least 10 mitotic cells were simultaneously followed and three independent experiments were conducted.

### Xenografts

The protocol was evaluated by the local ethic committee and was conducted by a habilitated researcher. *In-vivo* experiments were performed on four-week old female Swiss nude mice (Janvier, France). After one week of adaptation with their environment conditions, mice were injected subcutaneously with 3.5 × 10^6^ Mahlavu cells. Cells suspended in PBS were mixed 1:1 with cold growth factor free matrigel (Beckton Dikinson). Tumours were established after 7 days. At this time, the mice were randomly divided into 2 groups, which equalled in the mean tumour size of each group. Tumour volume was determined by measuring two perpendicular diameters using a clipper and then calculated as follows: V = 4/3 π d_1_^2^ × d_2_ in which d_1_ and d_2_ are the smallest and the largest diameter, respectively. One mice group (6 mice) received the treatment of C5M in NaCl 0.45% (100 μg compound per each 20 gr of mouse) whereas the control group (6 mice) was injected with NaCl 0.45% only. The compound or control buffer injections were carried out 4 times per week, stopped 1 day between 2 times. Totally, mice received 11 injections. The size of the tumour and the weight of each mouse were determined twice per week. Tumour growth was calculated by measuring the variations in volume during the drug or control treatment using the formula: (V_n_−V_0_)/V_0_. The mean values were determined by 6 mice, in each group.
